# Dipeptidyl Peptidase 4 Inhibition Ameliorates Chronic Kidney Disease in a Model of Salt-Dependent Hypertension

**DOI:** 10.1155/2019/8912768

**Published:** 2019-01-10

**Authors:** Donato Cappetta, Loreta Pia Ciuffreda, Anna Cozzolino, Grazia Esposito, Cristina Scavone, Luigi Sapio, Silvio Naviglio, Domenico D'Amario, Filippo Crea, Francesco Rossi, Liberato Berrino, Antonella De Angelis, Konrad Urbanek

**Affiliations:** ^1^Department of Experimental Medicine, Section of Pharmacology, University of Campania “Luigi Vanvitelli”, 80138 Naples, Italy; ^2^Department of Biochemistry, Biophysics and General Pathology, University of Campania “Luigi Vanvitelli”, 80138 Naples, Italy; ^3^Institute of Cardiology, Catholic University of the Sacred Heart, 00168 Rome, Italy

## Abstract

Cardiovascular diseases frequently coexist with chronic kidney disease that constitutes a major determinant of outcome in patients with heart failure. Dysfunction of both organs is related to chronic inflammation, endothelial dysfunction, oxidative stress, and fibrosis. Widespread expression of serine protease DPP4 that degrades varieties of substrates suggests its involvement in numerous physiological processes. In this study, we tested the effects of selective DPP4 inhibition on the progression of renal disease in a nondiabetic model of hypertensive heart disease using Dahl salt-sensitive rats. Chronic DPP4 inhibition positively affected renal function with a significant reduction in albuminuria and serum creatinine. DPP4 inhibition attenuated the inflammatory component by reducing the expression of NF-*κ*B, TNF*α*, IL-1*β*, IL-6, and MCP-1. Kidney macrophages expressed GLP-1R, and DPP4 inhibition promoted macrophage polarization toward the anti-inflammatory M2 phenotype. Finally, high degrees of NADPH oxidase 4 expression and oxidation of nucleic acids, lipids, and proteins were reduced upon DPP4 inhibition. Our study provides evidence of renoprotection by DPP4 inhibition in a nondiabetic hypertension-induced model of chronic cardiorenal syndrome, indicating that DPP4 pathway remains a valid object to study in the context of chronic multiorgan diseases.

## 1. Introduction

Multimorbid chronic diseases are an increasing burden on individuals, communities, and health care services. For example, the coexistence of chronic heart and kidney diseases, referred to as cardiorenal syndrome, has a recognized pathophysiological significance. The high prevalence of chronic kidney disease (CKD) in patients with heart failure (HF) and vice versa associates both organs' dysfunction to increased morbidity and mortality [1]. Most patients with HF and CKD share several risk factors, including hypertension, diabetes, obesity, and metabolic alterations [2–4]. CKD constitutes a major determinant of outcome in patients with HF with preserved ejection fraction (HFpEF), and common denominators, such as endothelial dysfunction, low-grade inflammation, and fibrosis, have been identified. Specific mechanistic pathways involved in these pathophysiological processes and organ crosstalk remain incompletely understood. Interestingly, recent findings indicate the important role of dipeptidyl peptidase 4 (DPP4) in the crosstalk between hepatocytes and adipose tissue in metabolic disease [5]. DPP4 is a membrane and soluble serine protease expressed by many cell types including renal tubular cells, endothelial cells, and T cells, and in many organs, such as the kidney, intestine, lung, pancreas, liver, spleen, and heart. Apart from glucagon-like peptide-1 (GLP-1), DPP4 degrades variety of bioactive substrates such as chemokines and neuropeptides suggesting its involvement in numerous physiological processes [6, 7]. Furthermore, since GLP-1 receptor (GLP-1R) distribution is not exclusive of the pancreas but has also been observed in the kidney, lung, and heart [8], the effects of DPP4 inhibition in extrapancreatic tissues may also be mediated by GLP-1. It is therefore possible that DPP4 activity has an influential role in chronic diseases that involve multiorgan damage. To this end, to determine whether DPP4 is involved in the progression of kidney pathology in a multiorgan disease settings, we used sitagliptin, a highly specific DPP4 inhibitor in Dahl salt-sensitive (Dahl/SS) rats, a nondiabetic model that embraces several features of a multimorbid chronic condition. Among gliptin class, sitagliptin was chosen for several reasons. It is a highly selective DPP4 inhibitor with a greater affinity for DPP4 over related serine peptidases such as DPP8 and DPP9. Sitagliptin has been the first gliptin introduced in the treatment of type 2 diabetes, and, to date, is the most commonly used medication of the class. Unlike other gliptins, i.e., saxagliptin and alogliptin, its use has not been associated with an increased risk of HF, particularly in patients who already have heart or kidney disease.

## 2. Materials and Methods

### 2.1. Animal Procedures

In this study, the animal research was in accordance to the National Ethical Guidelines (Italian Ministry of Health; D.L.vo 26, March 4, 2014), approved by the local ethics committee, and conformed to ARRIVE guidelines [9]. Experiments were performed in seven-week-old male Dahl/SS rats (Charles River Laboratories, Wilmington, MA, USA) that were maintained on a 12 h/12 h light/dark cycle in temperature- and humidity-controlled room. Animals were treated with a high-salt (HS) diet (8% NaCl) for 5 weeks to induce hypertension. Afterwards, rats continued the same HS diet and were randomly divided into 2 groups: sitagliptin-treated rats (*n* = 10; HS + SITA group; 10 mg/kg/day by oral gavage, reflecting human exposure levels) and vehicle-treated rats (*n* = 10; HS group). The control group (*n* = 10) were fed with a low-salt (LS) diet (0.3% NaCl, LS group). All the rats were sacrificed under anaesthesia with ketamine (100 mg/kg) and medetomidine (0.25 mg/kg) eight weeks later, and the blood, urine, and kidney tissues were collected. Serum (creatinine and blood urea nitrogen) and urine (creatinuria, albuminuria, and sodium and chloride excretion) samples were analyzed by the Abbott Lab Chemistry Analyzer ci 8200 (Abbott Park, IL, USA).

### 2.2. Sample Preparation

After abdominal aorta was cannulated, a perfusion with 10% phosphate-buffered formalin was performed. Finally, tissue was embedded in paraffin and histological sections of 5 *μ*m thickness were cut [10].

### 2.3. Histochemistry

Histological sections were deparaffinized with xylene and rehydrated with aqueous solutions of decreasing ethanol concentrations. Masson's trichrome staining (Sigma-Aldrich, St. Louis, MO, USA) was used to detect tissue fibrosis [11]. The extent of glomerulosclerosis was determined by counting sclerotic glomeruli and expressed as a percentage of sclerotic glomeruli over the total number of glomeruli. Intertubular fibrosis was measured morphometrically using 42-point ocular grid in 20 randomly selected fields and expressed as the area fraction. By means of immunofluorescence labelling and confocal microscopy, infiltration and phenotype polarization of macrophages were assessed by scoring cells labelled with antibodies against CD68 (Thermo Fisher Scientific, Waltham, MA, USA) and CD206 (R&D Systems, Minneapolis, MN, USA). The expression of GLP-1R (Novus Biologicals, Littleton, CO, USA), endothelial nitric oxide synthase (eNOS) (Thermo Fisher Scientific), and nuclear factor-*κ*B (NF-κB) (Santa Cruz Biotechnology, Dallas, TX, USA) was also evaluated. Peroxynitrite formation and its action on the proteins were assessed by nitrotyrosine detection (Merck Millipore, Milan, Italy) [12]. Oxidative damage was determined with 8-hydroxydeoxyguanosine (8-OHdG) (Trevigen, Gaithersburg, MD, USA) and 4-hydroxynonenal (4-HNE) (Abcam, Cambridge, UK) at nuclear and membrane level, respectively. The expression of reactive oxygen species- (ROS-) producing NADPH oxidase 4 (Nox4) (Abcam) was visualized. Nuclei were counterstained with DAPI or PI (Sigma-Aldrich). Fluorescein isothiocyanate- (FITC) and tetramethylrhodamine-5-(and 6)-isothiocyanate- (TRITC-) conjugated secondary antibodies were used (Jackson ImmunoResearch, Suffolk, UK). Sections were analyzed with a Leica DM5000B microscope (Leica Microsystems, Wetzlar, Germany) and a Zeiss LSM700 confocal microscope (Zeiss, Oberkochen, Germany). Nitrotyrosine, 8-OHdG, 4-HNE, and GLP-1R images were processed and analyzed by ImageJ for the quantification of fluorescence intensity, measured as the sum of pixel values in the channel of interest per unit area.

### 2.4. Western Blotting

Proteins were extracted with a lysis buffer containing 0.1% Triton X-100 and a cocktail of protease and phosphatase inhibitors (Sigma-Aldrich), and their concentration was measured by Bradford assay (Bio-Rad). The proteins were separated on (8%–12%) SDS-PAGE and transferred onto polyvinylidene fluoride membrane [13]. Membranes were probed with primary antibodies against transforming growth factor-*β* (TGF-*β*), TGF-*β* receptor (TGF-*β*R), SMAD3, tumor necrosis factor *α* (TNF*α*), interleukin 6 (IL-6), Nox2, Nox4, E-selectin (Abcam), NF-*κ*B, interleukin-1*β* (IL-1*β*), monocyte chemoattractant protein-1 (MCP-1) (Santa Cruz Biotechnology), eNOS (Thermo Fisher Scientific), and phospho-SMAD3^Ser423/425^ (Cell Signaling Technology, Danvers, MA, USA). Loading conditions were determined with glyceraldehyde 3-phosphate dehydrogenase (GAPDH) (Sigma-Aldrich). Peroxidase-conjugated secondary antibodies were employed to detect primary antibodies (Santa Cruz Biotechnology). Antibody binding was visualized by chemiluminescence, and images were collected and analyzed using a ChemiDoc-It Imager (Ultra-Violet Products, Cambridge, UK).

### 2.5. Data Analysis

Data were analyzed using GraphPad Prism (GraphPad Software, San Diego, CA, USA). The results are presented as mean ± SD, and the number of replicates was at least *n* = 5 per group for each data set. Data distribution was determined by a normality test. Significance among groups was determined by one-way ANOVA and Bonferroni's posttest. All *p* values are two-sided, and *p* < 0.05 was considered statistically significant.

## 3. Results

Treatment with sitagliptin had a beneficial impact on overall animal health and prevented weight loss. Levels of blood glucose were unchanged in all experimental groups excluding involvement of glycemic control in a pathological condition. During eight weeks of treatment, rats remained severely hypertensive and a significant reduction of blood pressure was only observed at 19 weeks of age. Thus, it is unlikely that this change could exclusively account for the beneficial effects of sitagliptin, although blood pressure reduction may have participated in functional and structural modifications. Finally, effective DPP4 inhibition was confirmed by the increase in levels of circulating GLP-1.

For a more detailed reading, refer to our previous publication [14].

### 3.1. Effect of DPP4 Inhibition on Renal Function

**Figure 1:**
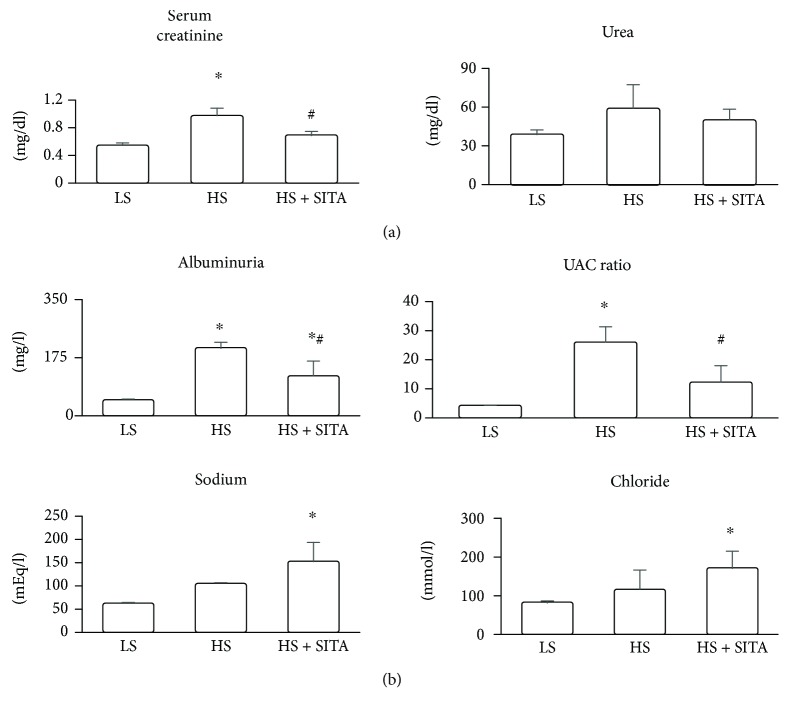
Blood and urine analysis. Renal function parameters assessed in blood (a) and urine (b) samples. Data represent the mean ± SD. ^∗^*P* < 0.05 vs. LS; ^#^*P* < 0.05 vs. HS.

In HS rats, serum creatinine was significantly elevated while blood urea showed a nonsignificant upwards trend (Figure 1(a)). Urine analysis (Figure 1(b)) revealed increased albuminuria with a striking elevation of urinary albumin-to-creatinine (UAC) ratio as compared to LS rats. Chronic DPP4 inhibition reduced albuminuria and UAC ratio, along with creatinine levels in serum, thus suggesting a protective effect on renal function. Urinary sodium and chloride concentrations were increased following DPP4 inhibition (Figure 1(b)).

### 3.2. Effect of DPP4 Inhibition on Renal Fibrosis

**Figure 2:**
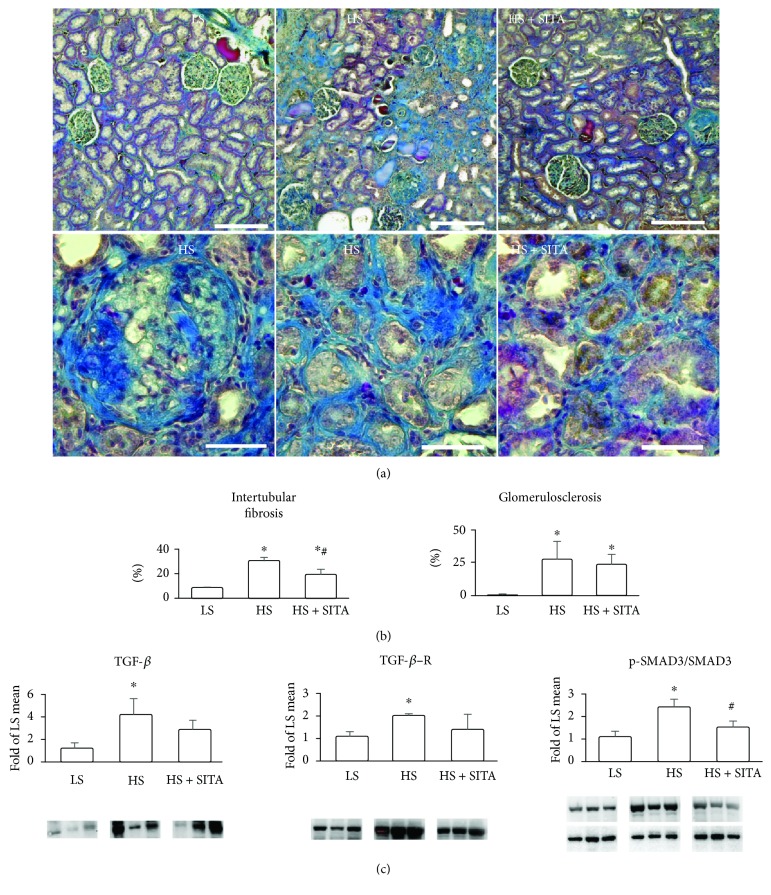
Renal fibrosis and profibrotic signaling. (a) Masson's trichrome staining showing collagen deposition (blue) in the kidneys of LS, HS, and HS + SITA animals. Lower panels are high-power photographs showing sclerotic glomerulus and intertubular fibrosis in HS and HS + SITA kidneys. (b) Area fraction occupied by intertubular fibrosis and the percentage of sclerotic glomeruli. (c) Protein expression of TGF-*β*, TGF-*β*R, and phospho-SMAD3^Ser423/425^/SMAD3 ratio. Scale bars: (a) upper panels 200 *μ*m, lower panels 50 *μ*m. Data represent the mean ± SD. ^∗^*P* < 0.05 vs. LS; ^#^*P* < 0.05 vs HS.

HS animals presented remarkable renal fibrosis. Masson's staining revealed significant glomerulosclerosis and the presence of severe tubulointerstitial fibrosis (Figure 2(a)). DPP4 inhibition reduced the extent of intertubular fibrosis while the fraction of sclerotic glomeruli remained unchanged (Figures 2(a) and 2(b)). Western blotting analysis showed the upregulation of TGF-*β* signaling induced by high-salt diet. Increased levels of TGF-*β* were accompanied by a consistent rise of TGF-*β*R and increased phosphorylation of SMAD3. Sitagliptin partially suppressed the TGF-*β* pathway with a significant reduction of phospho-SMAD3 expression indicating the amelioration of a profibrotic phenotype (Figure 2(c)).

### 3.3. Effect of DPP4 Inhibition on Inflammation

**Figure 3:**
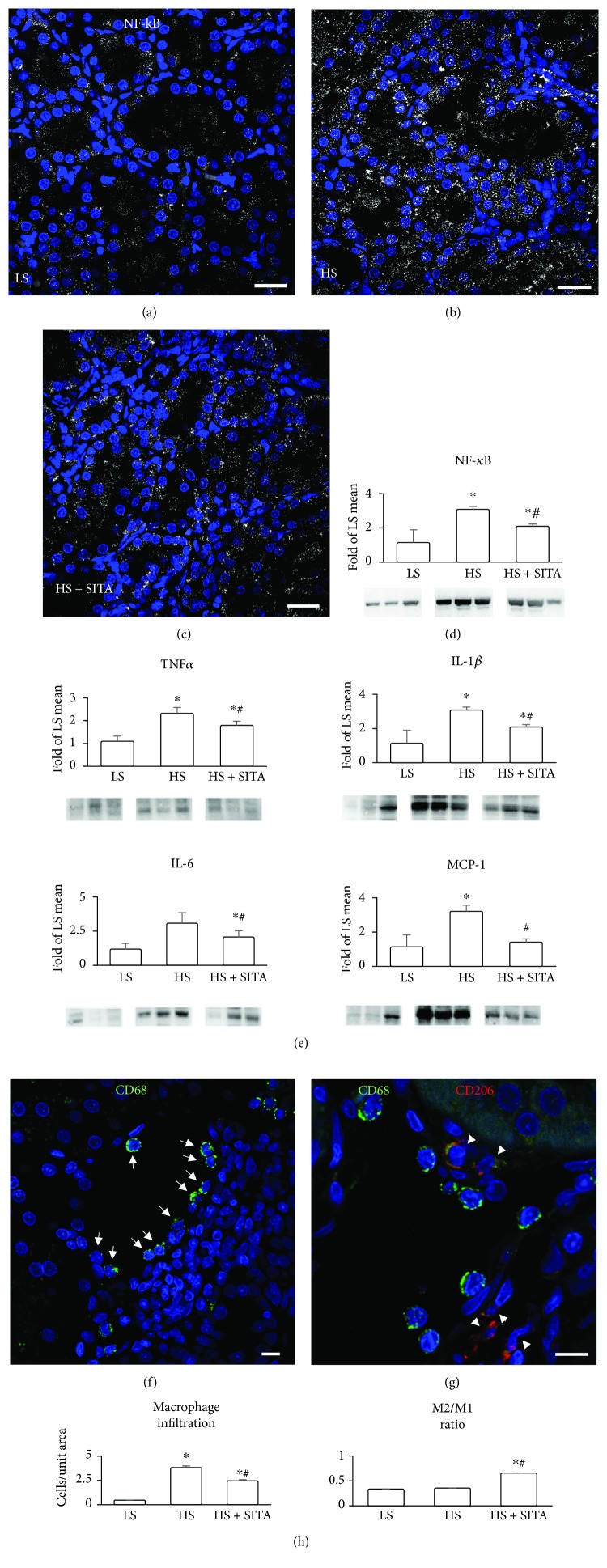
Proinflammatory signaling and macrophage phenotype. (a–c) Immunofluorescence showing the expression of NF-*κ*B (white, pseudocolor) in the tubules and interstitium in LS (a), HS (b), and HS + SITA (c) kidneys. (d) Protein expression of NF-*κ*B by western blot. (e) Renal TNF*α*, IL-1*β*, IL-6, and MCP-1 protein levels. (f) Infiltration by macrophages labelled by CD68 (green, arrows) in the kidney of HS rat. (g) Macrophages of M2 subtype identified by the expression of CD206 (red, arrowheads) in the HS + SITA kidney. Nuclei are stained with DAPI (blue). (h) Numerical density of tissue macrophages and the fraction of M2 macrophage subpopulation. Scale bars: (a–c) 25 *μ*m; (f, g) 10 *μ*m. Data represent the mean ± SD. ^∗^*P* < 0.05 vs. LS; ^#^*P* < 0.05 vs. HS.

Inflammation is a major component of hypertension-induced renal injury. Hence, signaling of NF-*κ*B, known to activate various proinflammatory genes, and infiltration of monocytes/macrophages, the main source of inflammatory mediators, were evaluated. Immunofluorescence visualized the expression of NF-*κ*B in tubular epithelium (Figures 3(a)-3(c)). Kidneys of HS rats showed increased signal in tubules and in interstitial cells. Expression of NF-*κ*B was detected and quantified also by western blot (Figure 3(d)). Increased levels of proinflammatory cytokines such as TNF*α*, IL-1*β*, IL-6, and chemokines such as MCP-1 were also present in HS animals. These inflammatory effectors were significantly downregulated by chronic DPP4 inhibition. In parallel, high-salt diet markedly increased the number of infiltrating macrophages, detected by CD68, mostly exhibiting CD206-negative proinflammatory M1 phenotype (Figure 3(e)). DPP4 inhibition reduced the number of infiltrating cells and promoted a phenotype switch to the anti-inflammatory M2 subtype (CD206-positive cells) (Figures 3(f) and 3(g)).

### 3.4. Involvement of GLP-1 Signaling

**Figure 4:**
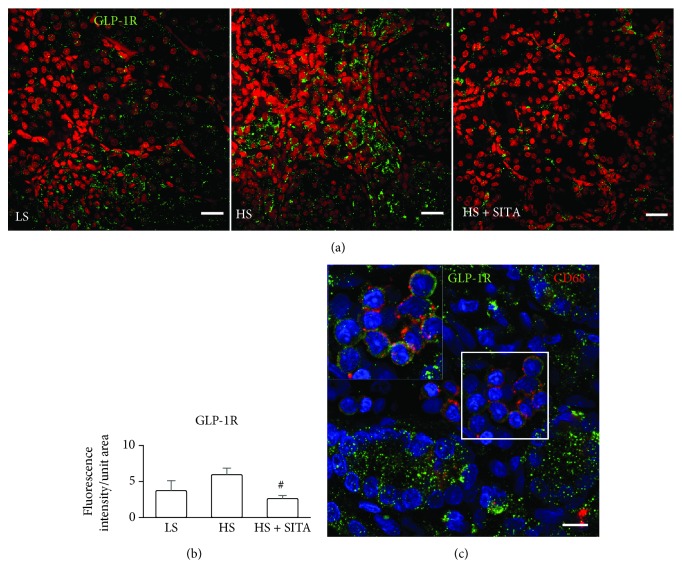
GLP-1 receptor. (a) Expression of glucagon-like peptide-1 receptor (GLP-1R, green) in the kidneys of LS (left panel), HS (central panel), and HS + SITA (right panel) animals. Nuclei are stained with PI (red). (b) Quantification of fluorescence intensity. (c) Cluster of macrophages expressing CD68 (red) and GLP-1R (green) in the renal tissue. The area in the square is shown at higher magnification in the inset. Nuclei are stained with DAPI (blue). Scale bars: (a) 25 *μ*m; (c) 10 *μ*m. ^#^*P* < 0.05 vs. HS.

The expression of GLP-1R has predominantly been detected in the tubular compartment and in monocytes/macrophages infiltrating the kidney (Figure 4). This indicates the involvement of GLP-1/GLP-1R axis in tubular function and the behavior of intrarenal inflammatory cells. Surprisingly, given a possible protective role mediated by the activation of GLP-1/GLP-1R system, the signal of GLP-1R was enhanced in the HS group as compared to that of the LS and HS + SITA rats.

### 3.5. DPP4 Inhibition and Endothelial Activation

**Figure 5:**
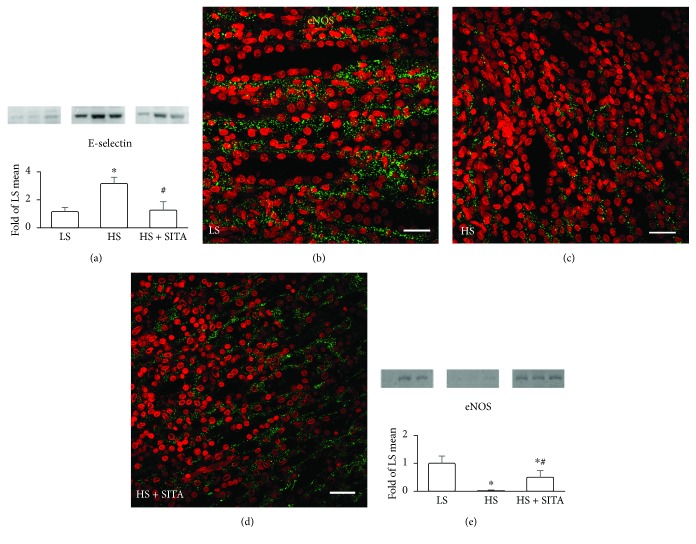
Endothelial activation and dysfunction. (a) Renal expression of E-selectin by western blot. (b–d) Immunofluorescence showing renal expression of eNOS (green) in LS (b), HS (c), and HS + SITA (d) rats. Nuclei were counterstained with PI (red). (e) Protein expression of eNOS. Scale bars: 25 *μ*m. ^∗^*P* < 0.05 vs. LS; ^#^*P* < 0.05 vs. HS.

We also evaluated the vascular endothelium, another key component in CKD. Endothelial dysfunction is elicited by inflammation and sustains inflammatory process by expressing adhesion molecules thus recruiting circulating immune cells. The protein content of E-selectin was found elevated in the HS group (Figure 5(a)), suggestive of activation of endothelial cells promoting immune cell attraction in the kidney. Indeed, the clusters of monocytes attracted to renal vessel endothelium were frequently seen in our study (see Figures 3(f) and 4(b)). The observed overexpression of E-selectin was downregulated with the DPP4 inhibition. Remarkably, in the kidneys of HS rats, the quantity of eNOS was reduced by 94%. This severe deficit was partly restored following the treatment with sitagliptin. Nearly 9-fold increase in eNOS levels, as compared with HS rats, strongly suggests the positive effects of DPP4 inhibition on endothelial function (Figures 5(b)–5(e)).

### 3.6. Effect of DPP4 Inhibition on Oxidative Damage

**Figure 6:**
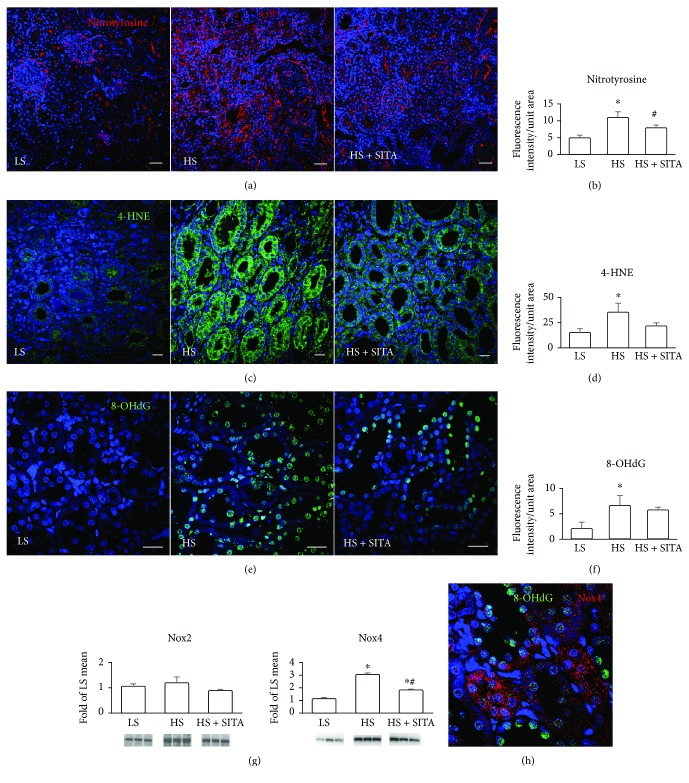
Oxidative and nitrative stress. Detection and fluorescence quantification of nitrated proteins (nitrotyrosine, red) (a, b); oxidized lipids, 4-hydroxynonenal (4-HNE, green) (c, d); DNA, 8-hydroxydeoxyguanosine (8-OHdG, green) (e, f) in the kidneys of LS (left panels), HS (central panels), and HS + SITA (right panels) rats. (g) Protein expression of NADPH oxidase isoforms, Nox2 and Nox4. (h) Tubular epithelial cells expressing Nox4 (red) show oxidative injury of DNA (8-OHdG, green). Nuclei are stained with DAPI (blue). Scale bars: (a) 50 *μ*m; (c, e, h) 20 *μ*m. ^∗^*P* < 0.05 vs. LS; ^#^*P* < 0.05 vs. HS.

The contribution of free radicals to the progression of renal damage was evidenced by the assessment of oxidative/nitrosative stress. In HS rats, detection of nitrotyrosine by immunofluorescence and confocal microcopy documented a high degree of protein nitration that is a result of the interaction of peroxynitrite with tyrosine residues of proteins (Figures 6(a) and 6(b)). Moreover, striking level of 4-HNE immunoreactivity evidenced intense membrane phospholipid peroxidation in the tubular epithelial cells (Figures 6(c) and 6(d)). Oxidative injury in the nuclear compartment with an increased formation of 8-OHdG foci documented extensive DNA damage (Figures 6(e) and 6(f)). These features of chronic oxidative injury of the kidney were diminished following DPP4 inhibition. Furthermore, the expression of Nox4, the main NADPH isoform to produce ROS in the renal compartment, resulted a marked increase in HS animals and significant downregulation in sitagliptin-treated rats. The levels of Nox2 were not changed in any experimental group (Figure 6(g)). We have observed that DNA damage marker 8-OHdG, predominantly present in the nuclei of tubular cells, was associated with the expression of Nox4 (Figure 6(h)).

## 4. Discussion

In this study, we tested the effects of selective DPP4 inhibition on the progression of renal disease in a nondiabetic model of hypertensive heart disease [15]. Dahl/SS rats, when fed a high-salt diet, develop hypertension that evolves into HFpEF, with left ventricle hypertrophy, diastolic dysfunction, high filling pressures, and lung congestion while maintaining ejection fraction preserved. They also show an increase of serum creatinine and proteinuria consequently to kidney injury. Evidence of inflammation, oxidative stress, and low nitric oxide bioavailability in the kidney defines the central importance of this organ and points to renal disease, along with HFpEF, as a key pathophysiological component [16]. Together with our previous results, the present study allows to view this model from a broader perspective. Dahl/SS rats experience diverse hallmarks of metabolic syndrome, such as hypertension and insulin resistance, which accompany heart and kidney damage. This multifactorial background may define Dahl/SS rat as a model of chronic disease that features cardiorenal syndrome. We show that a progressive raise of blood pressure, increased albuminuria, serum creatinine, and high UAC ratio was associated with prominent glomerular sclerosis and tubulointerstitial fibrosis. While the relationship between albuminuria and myocardial dysfunction is not completely understood, a whole-body phenomenon of endothelial dysfunction and chronic inflammation may be a link that associates disease manifestation at different sites [17]. In fact, in parallel to ongoing CKD, the heart shows alterations in diastolic function, rise in circulating brain natriuretic peptide, cardiomyocyte hypertrophy, and massive accumulation of fibrotic tissue [14, 18]. In both organs, these changes are related to enhanced inflammation, endothelial dysfunction, and oxidative stress. This multifaceted pathophysiologic profile resembles the complexity held by a large population of multimorbid patients with chronic diseases.

Up to date, specific mechanistic pathways involved in these pathophysiological processes and organ crosstalk remain incompletely understood, but very recent findings indicate the important role of DPP4 in communication between hepatocytes and adipose tissue in metabolic disease [5]. Our data follow this thread and reinforce the recognition of pleiotropic effects of DPP4 inhibition that can translate in renal benefits [7]. In addition to potential systemic action, renal expression of DPP4 and GLP-1R points to in situ effects driven by extrapancreatic GLP-1 signaling and DPP4 inhibition [19]. Upregulation of renal DPP4 has been demonstrated in both experimental CKD models and human glomerular disease [20] and has been correlated to glomerulosclerosis in diabetic nephropathy [21]. Clinical and experimental evidence shows that DPP4 inhibition may drive nephroprotective effects [22, 23]. Preclinical studies in diabetic animals [20, 24] have shown that DPP4 inhibitors reduce the level of urinary albumin excretion and exert a renoprotective effect. Positive effects were also reported in the absence of diabetes, although with gliptins with relatively low DPP4 selectivity [25, 26]. Also in diabetic patients, DPP4 inhibitor-dependent beneficial effects on renal function with suppressed albumin excretion or improved UAC ratio have been demonstrated [27–31]. In our work, eight-week treatment with sitagliptin positively affected the renal function. A significant reduction in albuminuria, UAC ratio, and serum creatinine was observed with respect to untreated hypertensive animals.

Several studies have correlated endothelial dysfunction and chronic low-grade inflammation to development of CKD, providing a broader mechanistic framework of cardiorenal disease [3, 32]. NF-*κ*B is a master regulator of the inflammatory process promoting the transcription of several proinflammatory genes including cytokines and adhesion molecules in the heart as well as in the kidney [33, 34]. Our data show the contribution of proinflammatory molecules in response to high-salt intake and hypertension. DPP4 inhibition attenuated the inflammatory component by reducing the expression of NF-*κ*B and its downstream effectors TNF*α*, IL-1*β*, IL-6, and MCP-1. This is consistent with numerous evidences showing that both incretin mimetics and DPP4 inhibitors exert anti-inflammatory action [24, 25, 35–37]. Proinflammatory cytokines, adhesion molecules, and chemoattractant proteins promote homing of inflammatory cells to the kidney [33, 38, 39]. High-salt loading increases renal macrophage infiltration, which amplifies the inflammatory response causing renal damage and vascular dysfunction and resulting in avid sodium retention and elevated vascular resistance [40–42]. It is worth taking into account that the anti-inflammatory effect of sitagliptin may interrupt the cytokine-related enhanced expression of the epithelial Na^+^ channel and Na^+^-Cl^−^ and Na^+^-K^+^-Cl^−^ cotransporters that regulate salt and water balance favoring Na^+^ excretion [42, 43].

Our data show that DPP4 inhibition reduced macrophage infiltration of the injured kidney and induced a marked macrophage polarization toward the anti-inflammatory M2 phenotype that secretes anti-inflammatory cytokines and participates to wound healing and tissue repair [44, 45]. The observed effect may be the result of GLP-1 pathway activation. Our findings showing kidney monocytes/macrophages expressing GLP-1R support the evidence of a similar effect on cell polarization exerted by incretin analogs [35, 46]. In addition, expression of GLP-1R has been described in several renal compartments, with a predominant presence in proximal tubular cells, where its activation inhibits tubular reabsorption and promotes urinary flow and sodium excretion [47]. Activation of GLP-1 pathway increased diuresis and natriuresis, likely by downregulation of Na^+^/H^+^ exchanger isoform 3 (NHE3), and reduced albuminuria resulting in improvement of renal damage, in both rodent models and humans [19, 22, 48, 49]. The increased natriuresis observed in HS + SITA animals that was accompanied by increased excretion rates of chloride can be a result of several processes. Moreover, as the anti-inflammatory effect is linked to different local cytokine profiles, it can influence on the expression of several ion transporters in the tubules [43]. Also, the decreased oxidative stress and increased NO may directly influence tubular Na and Cl reabsorption [50]. While the main effect of GLP-1 is stimulation of insulin secretion, local regulation of its receptor and catabolism may be crucial also at the kidney level. Indeed, DPP4 also is expressed by tubular cells where it catabolizes GLP-1 [51, 52]. Moreover, DPP4 is physically linked to NHE3 and can modulate Na^+^/H^+^ exchange [53]. Our data on the renal GLP-1R expression in HS animals and the increase in urine sodium concentration following DPP4 inhibition indicate a role of this system also in the pathobiology of hypertensive chronic kidney disease. Surprisingly, the expression of GLP-1R resulted an increase after high-salt loading and decrease in response to sitagliptin treatment. This data seems to be in conflict with the protective role of GLP-1/GLP-1R axis. One reasonable explanation could be that the kidney promotes the upregulation of GLP-1R in response to low level of circulating and local GLP-1 due to degradation by DPP4. Conversely, the inhibition of DPP4 and consequent increase of GLP-1 restores the “physiological” expression of the receptor.

Inflammation is strongly related to endothelial dysfunction when the decreased nitric oxide bioavailability restricts endothelial vasodilatory, antiadhesive, and anti-inflammatory properties. Our data show that endothelium also benefits from DPP4 inhibition by means of the increase in eNOS expression and the reduction in UAC ratio.

Oxidative stress and inflammation create a vicious loop that drives the progression of kidney disease. NADPH oxidases are main source of ROS that play a recognized role in development and progression of kidney disease [54–56]. Overexpression of the isoform Nox4 is associated with vascular and renal remodeling in Dahl rats [54, 57]. Consistently, in our experimental setting, the level of Nox4 was increased in the group under high-salt diet and reduced upon DPP4 inhibition. Reduced release of proinflammatory cytokines by macrophages may have contributed to the suppression of Nox4-dependent ROS production and attenuation of profibrotic TGF-*β*/SMAD3 signaling and kidney fibrosis. This is also in line with the evidence of the involvement of GLP-1 signaling, through protein kinase A, in reducing renal Nox4 expression and ROS accumulation [58]. High degree of oxidative injury observed in different cellular compartments with modified nucleic acids, lipids, and proteins highlights the fundamental role of oxidative burst in our experimental setting in which chronic DPP4 inhibition attenuated ROS/reactive nitrogen species-induced injury.

## 5. Conclusions

**Figure 7:**
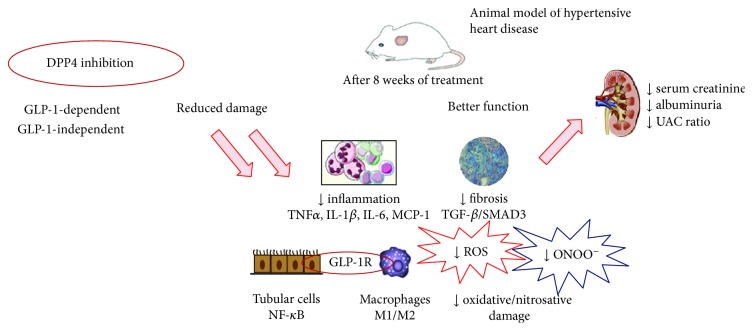
Schematic overview of mechanistic insights upon DPP4 inhibition.

This study provides evidence that DPP4 inhibition exerts renoprotective effects through anti-inflammatory actions in a nondiabetic, hypertension-induced model of cardiorenal syndrome. Suppression of macrophage infiltration and decrease in endothelial dysfunction and oxidative stress interrupt a vicious circle that feeds CKD progression (Figure 7). Thus, DPP4-related pathways remain a valid object of studies that can lead to innovative ways to face the problem of growing epidemic of chronic multiorgan diseases. The renoprotective properties of DPP4 inhibition have yet to be demonstrated convincingly in clinical trials. This may simply reflect the scarcity of studies designed to measure renal outcomes. For this reason, there is much interest in the results from the CARMELINA study (NCT01897532), a placebo-controlled clinical trial specifically designed to evaluate renal outcomes with linagliptin, in patients with type 2 diabetes at high risk for cardiovascular events.

## Data Availability

The data used to support the findings of this study are included within the article.
